# Stereotactic radiotherapy in neovascular age-related macular degeneration: Real-life efficacy and morphological evaluation of the outer retina-choroid complex: Erratum

**DOI:** 10.1097/MD.0000000000006856

**Published:** 2017-05-05

In the article “Stereotactic radiotherapy in neovascular age-related macular degeneration: Real-life efficacy and morphological evaluation of the outer retina-choroid complex”,^[1]^ which appeared in Volume 95, Issue 52, two of the authors’ degrees did not appear. Both Dirk Rades and Salvatore Grisanti's degrees should have appeared as “Prof.”
